# High Barrier Properties of Butyl Rubber Composites Containing Liquid Rubber and Graphene Oxide

**DOI:** 10.3390/nano14060534

**Published:** 2024-03-18

**Authors:** Jiaye Li, Zhanghao Yang, Shanjun Hu, Xianhong Huang, Stephen Jerrams, Shui Hu, Li Liu, Shipeng Wen

**Affiliations:** 1 State Key Laboratory of Organic-Inorganic Composites, Beijing University of Chemical Technology, Beijing 100029, China; 2 Zhongce Rubber Group Co., Ltd., Hangzhou 310000, China; 3 Faculty of Engineering and Built Environment, Technological University Dublin, D01 K8222 Dublin, Ireland

**Keywords:** butyl rubber, graphene oxide, liquid rubber, barrier properties

## Abstract

The high elasticity and excellent gas barrier properties of rubber composites make them irreplaceable in the field of sealing. Constructing a complicated barrier network to reduce free volume is crucial to improving gas barrier properties. In this research, liquid acrylonitrile-butadiene rubber/γ-Methacryloxypropyl trimethoxy silane (KH570) modified graphene oxide/butyl rubber composites (LNBR/KGO/IIR) were fabricated. A KGO lamellar network was constructed to resist gas diffusion in the IIR matrix. Meanwhile, LNBR macromolecules further occupied the free volume inside the IIR composites, thereby maximizing the retardation of the path of small molecule gas permeation. The modification of GO by KH570 was successfully demonstrated through FTIR and XRD. The grafting rate of KH570 was calculated to be approximately 71.4%. KGO was well dispersed in IIR due to emulsion compounding and the formation of lamellar networks. The 300% modulus, tensile strength and tear strength of KGO/IIR were improved by 43.5%, 39.1% and 14.8%, respectively, compared to those of the IIR composite. In addition, the introduction of LNBR resulted in a 44.2% improvement in the gas barrier performance of nitrogen permeability relative to the original IIR composite.

## 1. Introduction

High-performance barrier rubber composites have become indispensable in the structure of tire inner liners, medical plugs, chemical protection and aerospace applications due to their unique high elasticity and sealing properties [1,2,3]. Along with the broadening of application fields and the complexity and variability of application environments, ever higher requirements have been needed from barrier rubbers. Due to the dense side methyl groups, butyl rubber has excellent gas barrier performance, and is often used as the matrix material in gas barrier rubbers [4,5,6]. Therefore, it is a challenge to further improve the gas barrier performance of butyl rubber composites by constructing special structures of the composites. Gas molecules easily penetrate free volumes in rubber composites due to their high molecular chain flexibility. Therefore, the reduction of free volume is the key to improving gas barrier properties. Previous researchers mainly focused on the introduction of fillers to occupy the free volume in rubber composites, resulting in improvements in barrier properties. Theoretical calculations by Gruber et al. [7] showed that a high carbon black (CB) content, small CB size and the formation of CB aggregation are beneficial to enhancing the gas barrier properties of rubber composites. Raju et al. [8] investigated the effect of different structural CB on gas-tightness properties of BIIR/ENR50 rubber composites and found that this property was enhanced by 15% on the basis of the same filler amount when using low-structural CBs. The addition of traditional one-dimensional fillers has greatly reduced the permeability of the gas-liquid and improved the air-tightness properties of the composites. However, the dispersion and interfacial properties of traditional one-dimensional fillers still results in the rubber composites having large internal cavities which consequently limits the improvements in gas barrier properties [9,10].

In recent years, graphene oxide (GO), a two-dimensional lamellar filler with a high specific surface area, has played an excellent role in the creation of high-performance rubber composites [11,12]. The two-dimensional lamellar structure produced can form large interfaces with the rubber macromolecules, resulting in fewer cavities inside the rubber composites and enhanced gas tightness [13]. In addition, the lamellar network structure can deflect the permeation path of the gas, further increasing the complexity of the gas penetration path [14,15,16]. Yang et al. [17] introduced GO into bromobutyl rubber (BIIR) using the Pickering emulsion template method and constructed a two-dimensional large graphene oxide barrier wall. The gas-tightness of the BIIR was reduced by 91% compared to that of the original BIIR. Wang et al. [18] investigated a green method for the preparation of GO/acrylonitrile-butadiene rubber composites. The air permeability coefficient of the rubber composite was significantly reduced by approximately 56% through the formation of induced chemical cross-linking with GO in the absence of vulcanization components.

Even though construction of a two-dimensional lamellar network can ably block the permeation of gases in rubber composites, numerous cavities are still present in the rubber composites, providing opportunities for the diffusion and permeation of gases. Therefore, how to fill these cavities in the composites containing lamellar filler networks is still an interesting topic.

Liquid rubber, a low molecular polymer, can be added to rubber composites to improve the dispersion of fillers while toughening the rubber composites [19,20,21]. In addition, it should be noted that the low molecular weight of molecular chains of liquid rubber have the ability to fill the free volume in rubber composites [22,23], further enhancing the interaction with rubber molecules by physical entanglement or chemical cross-linking [24,25,26]. Therefore, the filling of liquid rubber has the possibility of reducing the gas permeability of rubber composites.

In this research, GO was first modified using γ-methacryloxypropyl trimethoxy silane (KH570) to prepare KGO before further mixing with butyl rubber (IIR) latex using emulsion compounding to obtain an IIR composite with a fine KGO dispersion and a strong interfacial bonding between KGO and IIR. Furthermore, liquid acrylonitrile-butadiene rubber (LNBR) was introduced to reduce the cavities present in the KGO/IIR rubber composites. The effects of KGO and LNBR on the air-tightness performance of LNBR/KGO/IIR are discussed in the following text.

## 2. Materials and Methods

### 2.1. Materials

Butyl rubber (IIR, 1953) was purchased from Shandong Chambroad Petrochemicals Co., Ltd., Binzhou, China. Butyl rubber latex (solid content: 55%) was purchased from Zhejiang Kaiao New Material Co., Ltd., Jiaxing, China. Liquid acrylonitrile-butadiene rubber (LNBR, molecular weight: 5000) was purchased from Hengshui Ryan rubber and plastic technology Co., Ltd., Hengshui, China. Carbon black (CB, N330) was purchased from Cabot Corporation Co., Ltd., Shanghai, China. Graphene oxide (GO, solid content: 1.06%) was purchased from The Sixth Element (Changzhou) Materials Technology Co., Ltd., Changzhou, China. Calcium chloride (CaCl_2_, AR) was obtained from Beijing HongHu LianHe HuaGong ChanPin Co., Ltd., Beijing, China. And γ-Methacryloxypropyl trimethoxy silane (KH570, AR) was provided by Beijing Yancheng Technology Co., Ltd., Beijing, China. Zinc oxide (ZnO), stearic acid (SA), 2,2′-Dithiobis(benzothiazole) (accelerant DM), 1,1′-dithiobis(n, n-dimethylthio-formamide) (accelerant TT), and sulfur were all commercially available.

### 2.2. Sample Preparation

#### 2.2.1. Preparation of KH570 Modified GO

First, the GO slurry was diluted with deionized water to form a 3 g/L GO solution. The GO solution was further ultrasonicated for 30 min at room temperature. Then, 1 L of GO solution was added into a three-necked flask and 6 g of KH570 was added drop by drop. The modification was completed after stirring the reaction for 5 h at 80 °C. 

#### 2.2.2. Preparation of LNBR/KGO/IIR Composites

Initially, 1.5 L of KGO solution was mixed with 55 g of butyl rubber latex in a beaker and mechanically stirred at room temperature for 30 min. Next, 5 wt% calcium chloride aqueous solution was poured into the beaker and stirred for 5 min to flocculate the KGO and IIR, and then the flocculated masterbatch was collected using centrifugation. The KGO/IIR masterbatch collected was washed several times and dried in an oven at 60 °C. For consistency, the GO/IIR masterbatch was also prepared according to the same procedure. 

Table 1 shows the formulation of rubber composites. The units of the values in Table 1 is the parts by weight per hundred-part rubber (phr). These raw materials were mixed on a two-roll mill to obtain the compounds. Then, the compounds were cured at 153 °C under a pressure of 15 MPa to obtain IIR, GO/IIR, KGO/IIR and LNBR/KGO/IIR composites.

### 2.3. Characterizations

#### 2.3.1. Fourier-Transform Infrared Spectroscopy (FTIR)

The chemical structures of the KH570, GO and KGO were determined by Fourier-transform infrared (FTIR, TENSOR27, Bruker Corporation, Karlsruhe, Germany) spectroscopy. The wavenumber ranged from 4000 to 500 cm^−1^ at a scanning resolution of 4 cm^−1^.

#### 2.3.2. X-ray Diffraction (XRD)

The exfoliation structures of the GO and KGO were characterized by X-ray diffraction (XRD, D/Max2500 VB2+/PC, Rigaku Corporation, Tokyo, Japan). The scan range was between 5 and 90° at a scan rate of 10° min^−1^.

#### 2.3.3. Thermal Analysis (TGA)

The weight loss of all samples was identified using a TGA-1 thermal gravimetric analyzer (Mettler-Toledo Co., Zurich, Switzerland). The TGA test was performed at a heating rate of 10 K/min under a nitrogen atmosphere.

#### 2.3.4. Transmission Electron Microscopy (TEM)

The filler dispersion of IIR composites was observed using G220 S-TWIN transmission electron microscopy (TEM, FEI Co., Hillsboro, OR, USA).

#### 2.3.5. Rotorless Cure Meters

The vulcanization performance of the rubber composites was investigated using a rotorless cure meter (M-3000AU, Gotech Testing Machines Co., Ltd., Dongguan, China). The test was performed at 153 °C.

#### 2.3.6. Nuclear Magnetic Resonance (NMR)

The crosslinking densities of the rubber composites were measured using nuclear magnetic resonance (NMR, VTMR20-010V-1, Suzhou Niumag Corporation, Suzhou, China) spectroscopy. The test was performed at 0.5 ± 0.05 T, 15 MHz, and 90 °C. 

#### 2.3.7. Rubber Processing Analyzer (RPA)

The filler network of the composites was characterized using a rubber-processing analyzer (RPA, RPA2000, Alpha Technologies Co., Bellingham, WA, USA). The tests were performed at 1 Hz and 60 °C.

#### 2.3.8. Dynamic Mechanical Thermal Analyzer (DMTA)

The dynamic mechanical properties of the rubber composites were investigated using a dynamic mechanical thermal analyzer (DMTA, VA3000, METRAVIB Corporation, Isere France). The samples were subjected to tensile loading under a deformation of 0.1%. The test temperature ranged from −80 to +80 °C, and the heating and cooling rates were 3 °C/min.

#### 2.3.9. Tensile Test

The static mechanical properties were tested using a CTM4104 tensile test machine (SANS, Shenzhen, China) according to the ISO 37:2011 standard.

#### 2.3.10. Gas Permeability Measuring Apparatus

The nitrogen permeability of the rubber composites was tested using a gas permeability measuring apparatus (VAC-V2, Jinan Languang Electromechanical Technology Co., Ltd., Jinan, China).

## 3. Results

### 3.1. Characterization of KGO

**Figure 1:**
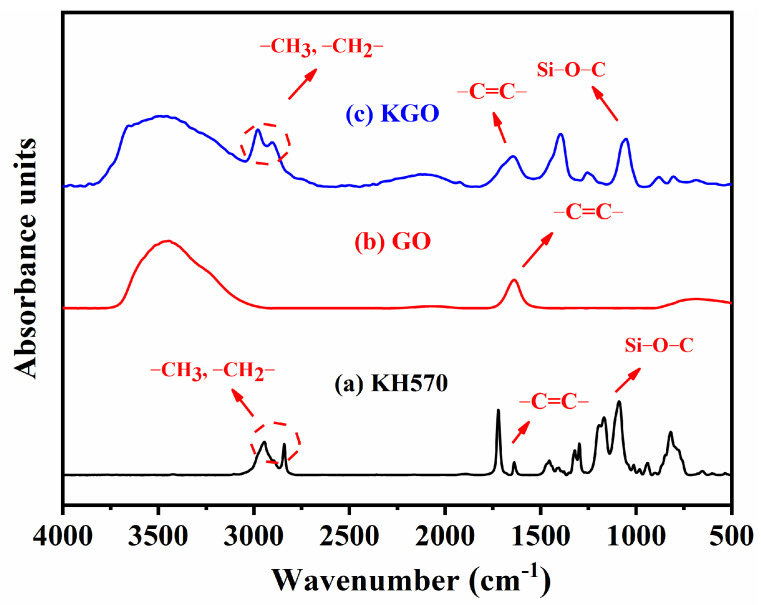
FTIR spectra of (**a**) KH570, (**b**) GO and (**c**) KGO.

Figure 1 shows the FTIR spectra of KH570, GO and KGO. Curve (a) for KH570 exhibits characteristic peaks of Si-O-C at 1080 cm^−1^ and -CH_3_ and -CH_2_ stretching vibration peaks at 2940 cm^−1^ and 2840 cm^−1^ [27,28]. Curve (b) for GO exhibits characteristic peaks of C=C at 1650 cm^−1^ and -OH stretching vibration at 3500 cm^−1^ [29]. After GO was modified with KH570, the silanol groups on the hydrolyzed KH570 reacted with the hydroxyl groups on the surface of GO to form Si-O-C covalent bonds. Curve (c) for KGO shows the stretching vibration absorption peaks of the Si-O-C bonds appearing at 1040 cm^−1^, and the -CH_3_ and -CH_2_ stretching vibration peaks of KH570 appeared at 2900 cm^−1^ and 2800 cm^−1^. Obviously, the C=C peak of KH570 at 1640 cm^−1^ in curve (a) shifts and combines with the C=C peak of GO at 1650 cm^−1^ in curve (b) and (c). The above changes indicate that KH570 successfully modified the GO.

**Figure 2:**
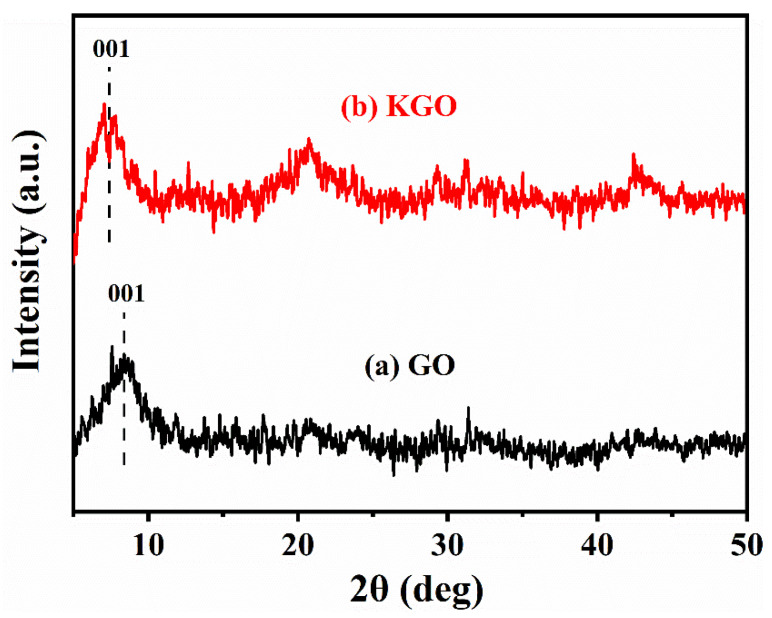
XRD patterns of (**a**) GO and (**b**) KGO powders.

Figure 2 shows the XRD patterns of GO and KGO. Curve (a) for GO exhibits the diffraction peak of the 001 crystal plane appearing at 8.4° [30], while the peak shifted to 7.3° after modification with KH570. According to the Bragg equation, the lamellar spacing of the KH570-modified GO became larger than that of GO, indicating that the KH570 successfully grafted onto the surface of GO and further exfoliated the GO sheets.

**Figure 3:**
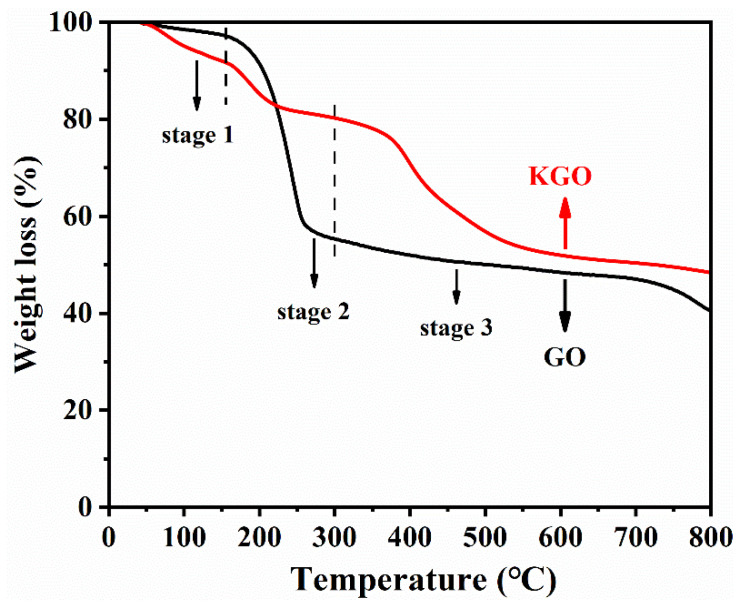
TGA curves of GO and KGO.

To further characterize the grafting rate and the thermal stability of GO, TGA curves of GO and KGO were obtained and are depicted in Figure 3. The GO curves mainly underwent three stages of thermal weight loss. Firstly, a slight weight loss in the range of 40–150 °C was attributed to the removal of a small amount of bound water between the GO sheets. Secondly, a large weight loss of about 42.1% occurred in the range of 150–300 °C due to the gradual removal of the oxygen-containing functional groups on the surface of GO at high temperatures. Thirdly, a small amount of weight loss in the range of 300–800 °C was attributed to rearrangement of the unstable carbocyclic compound structure and decomposition of the carbon skeleton at high temperatures [31]. The weight loss of GO reached 59.4% over the whole test temperature range.

KGO showed a weight loss of about 8% in the first stage due to the large spacing of the KGO sheets, which made it easier to adsorb the bound water. In the second stage, only 9.7% of weight loss was attributed to the elimination of a large number of oxygen-containing functional groups through the grafting reaction of KH570 on the surface of GO sheets. Based on the weight loss rates of GO and KGO in the second stage, the grafting rate of KH570 was calculated to be approximately 71.4%.

### 3.2. Characterizations of GO/CB/IIR Composites

**Figure 4:**
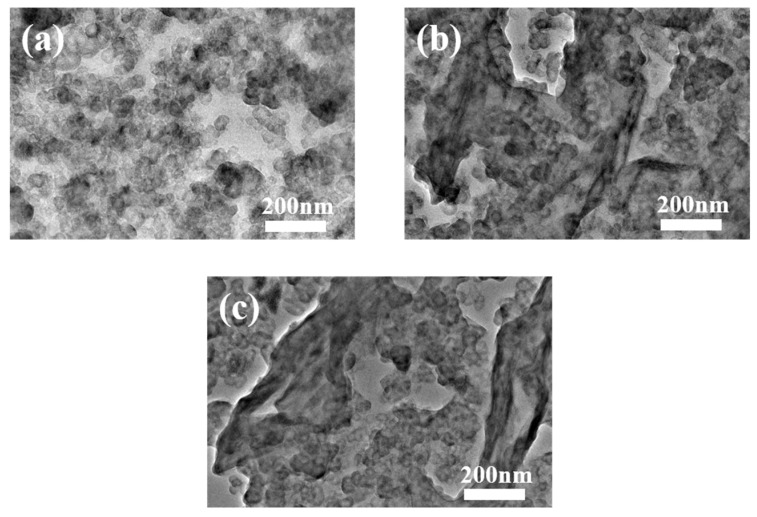
TEM images of (**a**) IIR, (**b**) GO/IIR and (**c**) KGO/IIR composites.

Figure 4 shows the TEM images of different rubber composites. Figure 4a shows that CB particles were prone to agglomeration and were poorly dispersed in the IIR matrix. After the addition of GO, Figure 4b shows that the GO strips dispersed between the CB particles, promoting the dispersion of CB and forming the complex filler network. For the KGO/IIR composite, Figure 4c shows that the KGO strips became broad and flat compared with GO in the rubber matrix. KGO also isolated the CB aggregate, leading to improved filler dispersion [32,33].

**Figure 5:**
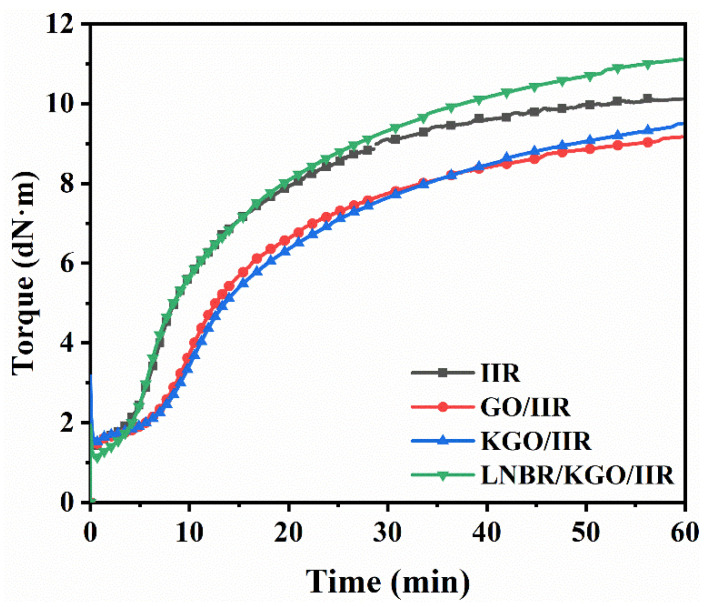
The vulcanization curves of IIR composites.

The abundant functional groups on the surface of GO can generate free radicals and participate in the cross-linking of rubber during vulcanization, leading to improvement in the cross-link density of the rubber composites [18,34]. The more complete network enhances the interfacial interactions between GO and rubber molecules, improving the barrier properties for gases. Figure 5 and Table 2 show the vulcanization performance of the rubber composites. The vulcanization rate of the rubber composites decreased after the addition of GO, attributed to the strong adsorption of sulfur and accelerated by the GO sheets [35,36]. After the addition of LNBR, the vulcanization rate of the rubber composite was accelerated, ascribed to the high activity of the LNBR and the high content of C=C double bonds.

**Figure 6:**
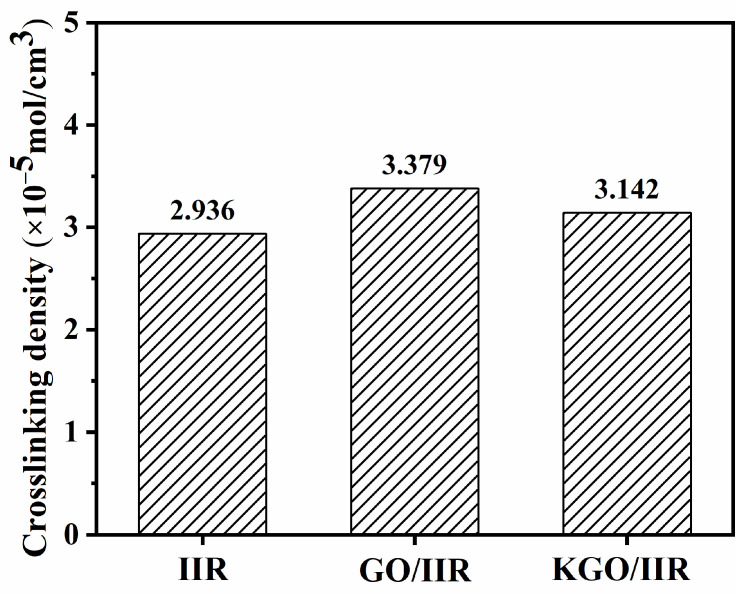
The crosslinking densities of IIR composites.

Figure 6 shows the crosslink densities of different IIR composites. Compared with the IIR composites, GO/IIR composites have a higher crosslink density. After the addition of KGO, the crosslink density of KGO/IIR composites decreased. This was because the silane coupling agent attached to the surface of GO may have hindered the crosslinking reaction between the free radicals generated by GO with the rubber [37]. However, the introduction of a silane coupling agent improves the GO dispersion as more interfacial layers formed between the GO and the rubber matrix, contributing to the airtightness of the composites [38,39].

**Figure 7:**
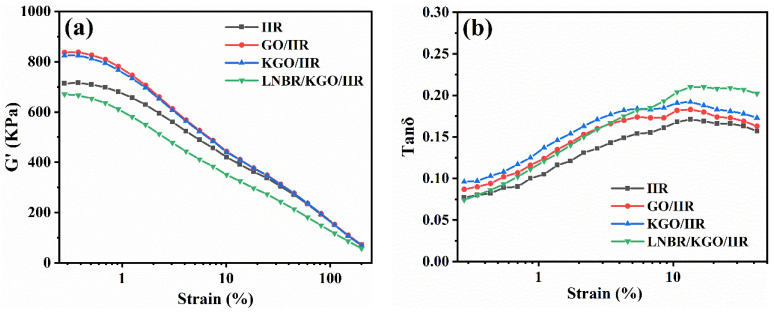
(**a**) Storage modulus–strain curves; (**b**) Loss faction–strain curves of IIR composites.

To characterize the effect of LNBR and GO on the interfacial strength of the rubber composites, the storage modulus and the loss factor of the rubber composites were evaluated as shown in Figure 7. Figure 7a shows the storage modulus of the rubber composites increased after the addition of GO or KGO, which can be attributed to the formation of strong interfacial interactions between the GO/KGO and IIR molecules [40]. However, the storage modulus of the IIR decreased after the addition of LNBR, which can be attributed to the plasticizing effect of LNBR.

Figure 7b shows that the loss factor of KGO/IIR was greater than that of the GO/IIR rubber composites, indicating that there was more internal friction within the KGO/IIR rubber composites during the dynamic deformation process, which can be attributed to weaker interfaces formed between KGO and IIR molecules. However, the added LNBR fully penetrated and filled the cavities in the IIR composite, resulting in less internal friction and a lower loss factor at small strains. At large strains, more LNBR contributed to dynamic movement, leading to an increase in the internal friction and a high loss factor of the composites.

**Figure 8:**
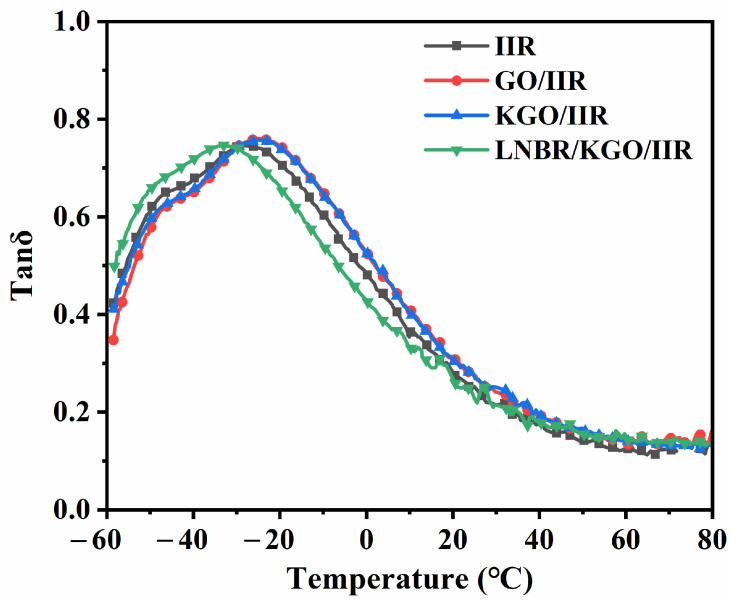
Loss factor–temperature curves of IIR composites.

To further analyze the effect of GO and LNBR on the rubber network structure, DMA tests were carried out. Figure 8 shows that the addition of GO or KGO enhanced the network structure of the rubber composites and the glass transition temperature shifted to the high temperature region. The increase in loss factor also further indicated the construction of a heightened interface between GO and the rubber matrix. After the addition of LNBR, the decrease in the glass transition temperature of the rubber composites indicated that LNBR penetrated the matrix of the rubber composites, resulting in a reduction in friction between the IIR molecular chains.

**Figure 9:**
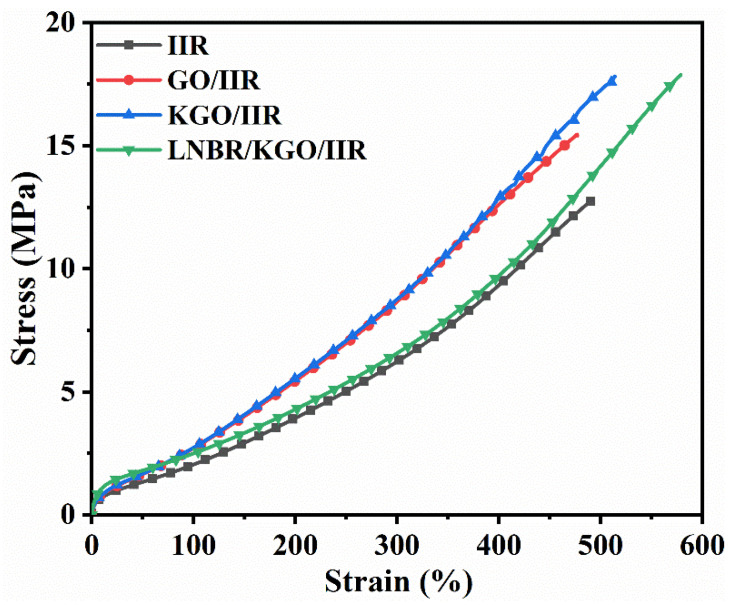
Stress–strain curves of IIR composites.

The mechanical properties of different rubber composites are shown in Figure 9 and Table 3. Compared to IIR, the rubber composites with GO and KGO exhibited improved mechanical properties. The 300% modulus, tensile strength and tear strength of KGO/IIR were improved by 43.5%, 39.1% and 14.8%, respectively, compared to those of the IIR composite. Compared with GO/IIR, the mechanical properties of KGO/IIR were further improved, which can be attributed to improved dispersion of KGO in the IIR matrix and more interfacial interactions between KGO and the IIR molecules. After the addition of LNBR in the KGO/IIRcomposites, LNBR molecules took some of the applied load, leading to an increase in the elongation at break. Moreover, LNBR, as a short molecular chain, also filled cavities which would have the effect of reducing the stress concentration around the cavities. Also, the plasticizing effect of LNBR reduced the 100% and 300% modulus of the IIR composites. 

**Figure 10:**
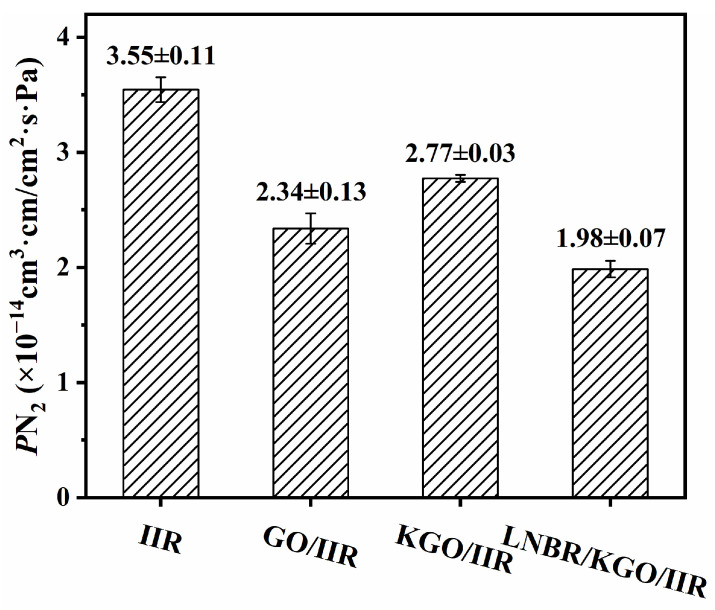
Nitrogen barrier properties of IIR composites.

Figure 10 shows the nitrogen barrier properties of different rubber composites. Due to the high barrier effect of GO sheets with large surface areas and good dispersion in the rubber matrices [38], the nitrogen permeability of GO/IIR and KGO/IIR rubber composites decreased by 34.0% and 21.9%, respectively, compared with that of IIR composites. After the further addition of LNBR, the cavities inside the IIR composites may have been filled by LNBR, which has a small molecular size, resulting in a further decrease of 44.2% in the nitrogen permeability of LNBR/KGO/IIR, compared to IIR.

**Scheme 1:**
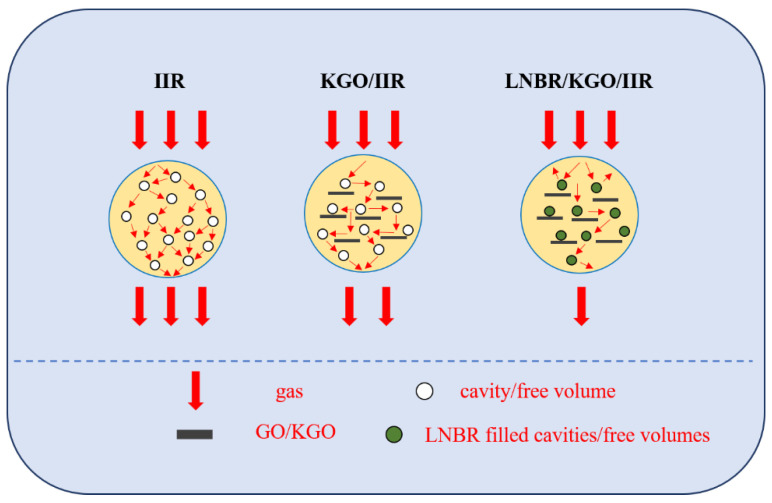
Gas permeation path in the IIR composites.

The schematic representation in Scheme 1 shows the gas barrier mechanism of the LNBR/KGO/IIR composites. For the IIR composite, gas rapidly penetrated and diffused through the internal cavities of the rubber. After the addition of GO, the layered filler network formed a tortuous path (Figure 4b) and hindered the diffusion of gas, resulting in the decrease in the nitrogen permeability (Figure 10) [41]. However, the presence of internal cavities can still cause gas permeation and diffusion. After the LNBR was introduced, it may have filled the cavities and reduced the free volume inside the IIR composites, making it difficult for gas molecules to penetrate, leading to the further decrease in the nitrogen permeability. Therefore, LNBE/KGO/IIR composites exhibit enhanced gas barrier performance.

## 4. Conclusions

In this research, KGO was obtained by modifying GO using the silane coupling agent KH570. A KGO/IIR masterbatch, with fine filler dispersion, was prepared using the emulsion compounding method. KGO formed a wider interface with the IIR molecular chain. The lamellar networks in the KGO/IIR composites also led to the enhancement of air-tightness performance by 21.9% compared with the IIR rubber composites. Thereafter, LNBR was introduced into the KGO/IIR to fill the cavities inside the rubber composites, leading to an improvement in the gas-tightness performance by 44.2% when compared with the IIR. The existence of cavities in the rubber composite not only provided a place for gas permeation, but also served as a place for crack propagation in the rubber composite, which adversely affected the dynamic performance of the rubber composite. The introduction of liquid rubber provides the possibility of solving cavity defects in these rubber composites, presenting a new strategy for improving the airtightness properties of rubber composites.

## Figures and Tables

**Table 1:** Formulation of rubber composites.

Samples	IIR	GO/IIR	KGO/IIR	LNBR/KGO/IIR
IIR	70	70	70	70
IIR Latex	30	30	30	30
GO	-	1.5	1.5	1.5
KH570	-	-	3	3
N330	40	40	40	40
ZnO	4	4	4	4
SA	2	2	2	2
accelerant DM	1.5	1.5	1.5	1.5
accelerant TT	0.8	0.8	0.8	0.8
S	2	2	2	2
LNBR	-	-	-	10

**Table 2:** The vulcanization characteristics of IIR composites.

Samples	MH (dN·m)	ML (dN·m)	ΔM (dN·m)	T10 (min)	T90 (min)
IIR	9.93	1.36	8.57	4.5	28.6
GO/IIR	8.99	1.43	7.56	6.6	39.8
KGO/IIR	9.31	1.48	7.83	7.2	43.0
LNBR/KGO/IIR	10.89	1.10	9.79	4.4	39.2

**Table 3:** Mechanical properties of IIR composites.

Samples	Shore a Hardness	100% Modulus (MPa)	300% Modulus (MPa)	Tensile Strength (MPa)	Elongation at Break (%)	Tear Strength (kN/m)
IIR	62 ± 1	2.0 ± 0.1	6.2 ± 0.2	12.8 ± 0.4	491 ± 11	34.5 ± 1.3
GO/IIR	64 ± 1	2.7 ± 0.2	8.6 ± 0.3	15.4 ± 0.5	477 ± 14	38.1 ± 1.5
KGO/IIR	65 ± 1	2.7 ± 0.1	8.9 ± 0.4	17.8 ± 0.5	514 ± 17	39.6 ± 1.7
LNBR/KGO/IIR	61 ± 1	2.5 ± 0.1	6.5 ± 0.2	17.8 ± 0.6	578 ± 16	46.2 ± 1.6

## Data Availability

Data are contained within the article.
